# Understanding altered contractile properties in advanced age: insights from a systematic muscle modelling approach

**DOI:** 10.1007/s10237-022-01651-9

**Published:** 2022-11-06

**Authors:** Dean L. Mayfield, Neil J. Cronin, Glen A. Lichtwark

**Affiliations:** 1 Department of Evolution, Ecology, and Organismal Biology, University of California, Riverside, Riverside, USA; 2 Neuromuscular Research Centre, Faculty of Sport and Health Sciences, University of Jyväskylä, Jyväskylä, Finland; 3 School of Sport and Exercise, University of Gloucestershire, Cheltenham, UK; 4 School of Human Movement and Nutrition Sciences, University of Queensland, Brisbane, Australia

**Keywords:** Ageing, Twitch, Force-frequency relationship, Calcium sensitivity, Specific force, Calcium uptake and release

## Abstract

Age-related alterations of skeletal muscle are numerous and present inconsistently, and the effect of their interaction on contractile performance can be nonintuitive. Hill-type muscle models predict muscle force according to well-characterised contractile phenomena. Coupled with simple, yet reasonably realistic activation dynamics, such models consist of parameters that are meaningfully linked to fundamental aspects of muscle excitation and contraction. We aimed to illustrate the utility of a muscle model for elucidating relevant mechanisms and predicting changes in output by simulating the individual and combined effects on isometric force of several known ageing-related adaptations. Simulating literature-informed reductions in free Ca^2+^ concentration and Ca^2+^ sensitivity generated predictions at odds qualitatively with the characteristic slowing of contraction speed. Conversely, incorporating slower Ca^2+^ removal or a fractional increase in type I fibre area emulated expected changes; the former was required to simulate slowing of the twitch measured experimentally. Slower Ca^2+^ removal more than compensated for force loss arising from a large reduction in Ca^2+^ sensitivity or moderate reduction in Ca^2+^ release, producing realistic age-related shifts in the force-frequency relationship. Consistent with empirical data, reductions in free Ca^2+^ concentration and Ca^2+^ sensitivity reduced maximum tetanic force only slightly, even when acting in concert, suggesting a modest contribution to lower specific force. Lower tendon stiffness and slower intrinsic shortening speed slowed and prolonged force development in a compliance-dependent manner without affecting force decay. This work demonstrates the advantages of muscle modelling for exploring sources of variation and identifying mechanisms underpinning the altered contractile properties of aged muscle.

## Introduction

Evidence has mounted in favour of the view that the intrinsic contractile properties of skeletal muscle are altered in advanced age. Age-related deficits in single fibre specific tension and maximum velocity of shortening have been found for type I and type II fibres from aged muscles of both rodents (Degens et al. 1998; Thompson et al. 1998; González et al. 2000, 2003; Zhong et al. 2006; Kim and Thompson 2013) and humans (Larsson et al. 1997; Frontera et al. 2000; Krivickas et al. 2001; D’Antona et al. 2003; Ochala et al. 2007; Yu et al. 2007; Lamboley et al. 2015; Power et al. 2016; Brocca et al. 2017) and can manifest despite long-term training (Korhonen et al. 2006; Power et al. 2016). Such findings imply that the broad decline in contractile performance with ageing isn’t the sole product of reductions in muscle fibre number and size. Reinforcing this notion are a more limited number of studies demonstrating that fundamental processes involved in activation and contraction are prone to impairment in old muscle, including the kinetics of cross-bridge cycling (Höök et al. 2001; D’Antona et al. 2003; Miller et al. 2013), mechanics of myosin (Lowe et al. 2001) and Ca^2+^ sensitivity of force (Brooks and Faulkner 1994; Lowe et al. 2002; Lamboley et al. 2015; Straight et al. 2018; Mazara et al. 2021), and handling of Ca^2+^ by the sarcoplasmic reticulum [SR (Larsson and Salviati 1989; Delbono et al. 1995; Narayanan et al. 1996; Wang et al. 2000; Jiménez-Moreno et al. 2008; Andersson et al. 2011; Umanskaya et al. 2014)].

Impaired intrinsic contractile performance, however, is not universally observed for old muscle (Trappe et al. 2003; Hvid et al. 2011; Sundberg et al. 2018; Teigen et al. 2020; Mazara et al. 2021). The same is true of disturbances to cellular level contractile processes, such as Ca^2+^ sensitivity of force (Eddinger et al. 1986; Plant and Lynch 2001; Lamboley et al. 2015; Teigen et al. 2020) and SR Ca^2+^ uptake (Fitts et al. 1984; Narayanan et al. 1996; Thomas et al. 2010). When human single fibre data published within the last decade are considered (Claflin et al. 2011; Hvid et al. 2011, 2017; Miller et al. 2013; Sundberg et al. 2018; Straight et al. 2018; Gries et al. 2019; Teigen et al. 2020; Grosicki et al. 2021; Mazara et al. 2021), a compelling argument could be made that neither type I nor type II fibres show an appreciable decline in specific force or shortening speed. Yet, age-related deficits in joint-level contraction speed and mass-specific mechanical power arise in the absence of impaired single fibre function (Reid et al. 2012; Sundberg et al. 2018). Prolonged or slowed force rise and decay and elevated force generation at submaximal stimulation frequencies (i.e. leftward-shifted force-frequency relationship) are also among the most commonly observed features of whole muscle in advanced age (Fitts et al. 1984; Davies et al. 1986; Larsson and Edström 1986; Vandervoort and McComas 1986; Brooks and Faulkner 1988; Alway 1995; Roos et al. 1999; Dow et al. 2005; McNeil et al. 2007; Tevald et al. 2009). A plausible explanation may be that an age-related elevation of the fractional area occupied by type I fibres or of the myosin heavy chain (MHC) I fibre content (Larsson et al. 1978; Coggan et al. 1992; Hunter et al. 1999; Short et al. 2005; Cui et al. 2008; Nilwik et al. 2013; Sonjak et al. 2019) is sufficient to produce a slower contractile phenotype (Ranatunga and Thomas 1990; Harridge et al. 1996).

Predicting altered mechanical output and identifying the underlying determinants remains challenging because contractile properties present inconsistently in advanced age, which may be reconciled with the myriad alterations that aged muscle can exhibit. Reports of the effect of age on single fibre or whole muscle contractile performance can be conflicting or show variation across taxa (Ballak et al. 2014), rodent strains (Rice et al. 2005), muscles (Brooks and Faulkner 1988; Brown and Hasser 1996; Narayanan et al. 1996; Hill et al. 2020), and as a function of activity level or training status (Fitts et al. 1984; Klitgaard et al. 1989; D’Antona et al. 2007), sex (Degens et al. 1998; Krivickas et al. 2001; Hill et al. 2020) and fibre type (Yu et al. 2007; Kim and Thompson 2013; Lamboley et al. 2015). Even for a given muscle of a model organism, the effect of ageing on contractile behaviour can vary (Brooks and Faulkner 1988; Moran et al. 2005). In addition to the aforementioned adaptations of cellular level function (e.g. slower cross-bridge kinetics), and a relative increase in type I fibre content, aged muscle may exhibit structural adaptations, such as altered intramuscular and extramuscular connective tissue properties (Gao et al. 2008; Wood et al. 2011; Stenroth et al. 2012; Danos et al. 2016; Holt et al. 2016). Compared to a loss of muscle mass, it is less clear how these adaptations (and others) impact contractile performance, especially when acting in concert, and to what extent these adaptations must present to be meaningful.

Determining the impact of age-related changes in muscle structure and function on mechanical output isn’t always feasible. For example, experimental approaches to quantifying excitation-SR Ca^2+^ release coupling and SR Ca^2+^ uptake dynamics may preclude myosin-actin interaction or be performed without simultaneous measurement of contractile force (Larsson and Salviati 1989; Delbono et al. 1995; Narayanan et al. 1996; Wang et al. 2000). In this context, it is also worth noting that crude homogenates of muscle frequently used to study Ca^2+^ release and Ca^2+^ uptake in advanced age (Fitts et al. 1984; Hunter et al. 1999; Thomas et al. 2010; Russ et al. 2011) may also be sensitive to an age-related increase in type I fibre content. The multifaceted and diverse nature of muscle deterioration and remodelling in response to ageing places importance on the interaction of adaptations. Interaction effects may be nonintuitive and may not always result in obvious impairment. Whereas an age-related reduction in the Ca^2+^ sensitivity of force may compound a reduction in SR Ca^2+^ release, an age-related slowing of SR Ca^2+^ uptake may offer a buffering effect. Linking any single adaptation to impaired contractile performance may be difficult when the scope of the study from an explanatory point of view is narrow and the broader extent of senescence is uncertain.

Establishing the likelihood that an altered property would appreciably impair contractile performance, in isolation and when acting in concert, might aid our understanding of altered contractile performance in advanced age from mechanistic and predictive perspectives. Muscle models are useful tools for exploring the effects of muscle design and adaptation on contractile performance (Wisdom et al. 2015). Several common traits of aged muscle contractile performance have been accurately simulated by adjusting model parameters to reflect known changes in activation and contraction dynamics and muscle–tendon morphology (Thelen 2003; Hasson and Caldwell 2012). Hill-type models simulate contractile behaviour according to well-established intrinsic mechanical phenomena (Curtin et al. 1998; Williams et al. 1998; Wakeling and Johnston 1999) and can be integrated with relatively simple, yet physiologically-grounded activation dynamics (Lichtwark and Wilson 2005a). Because many of the parameters used in Hill-type models can be related to muscle–tendon structure and intrinsic muscle properties, such models have the potential to elucidate the predominant mechanisms of altered force output and help explain unexpected observations and variance reported in the literature.

In this study, we implement a three-element Hill-type muscle model to examine how known changes in muscle function and structure in advanced age affect the contractile properties of muscle during fixed-end contractions. Drawing upon published literature, we simulate the individual and combined effects of impaired Ca^2+^ release, slower Ca^2+^ uptake, lower Ca^2+^ sensitivity of force, slowed intrinsic shortening speed, altered series elastic compliance, and a greater fractional content of type I fibres. Specifically, we evaluate the effects of these adaptations on isometric force during a twitch, brief tetanic contraction, and sustained contractions at submaximal and maximal stimulation frequencies. We then discuss the use of the model to explain how these known adaptations might affect muscle force, consider the adaptations most consistent with the contractile properties of aged muscle observed experimentally and reported by others in the literature, and identify certain conditions that may result in non-intuitive outcomes.

## Methods

### Experimental twitch data

Plantar flexion twitch torque was measured in 10 young (mean ± SD; age: 28 ± 3 years; body mass: 78 ± 11 kg; height: 179 ± 6 cm) and 18 older (age: 72 ± 5 years; body mass: 76 ± 10 kg; height: 174 ± 5 cm) healthy human adult males. An analysis of the experimental data obtained from young adults and a detailed description of the experimental protocol used for both young and older adults have been published previously (Mayfield et al. 2015). In brief, participants sat with their knee extended and right foot securely fixed to a non-compliant rotational footplate. The ankle was set to a neutral position (foot 90° relative to tibia). Two custom-built strain gauges positioned directly under the footplate measured isometric plantar flexion force evoked by percutaneous electrical stimulation of the tibial nerve. Single supramaximal square-wave pulses were delivered to elicit unpotentiated twitches, which were evaluated for peak torque, contraction time and half-relaxation time. Unpaired Student’s *t*-tests or Welch’s *t*-tests (unequal variance) were performed to test the effect of age. Statistical significance was set at *P* < 0.05 and effect sizes were calculated as eta squared (*η*^2^).

### Hill-type muscle model

We implemented an adapted Hill-type muscle model previously shown to successfully predict the time course of muscle force during contractions involving either ramp shortening or lengthening, or sinusoidal length changes (Curtin et al. 1998; Lichtwark and Wilson 2005a). The model (Mayfield and Lichtwark 2022), developed in Simulink (MathWorks, Natick, MA), consists of a contractile element (CE) and parallel elastic element (PEE) arranged in-series with an elastic element (SEE). The active force output of the CE depends on the interaction of CE activation, length, and velocity dynamics.

#### Activation

**Fig. 1:**
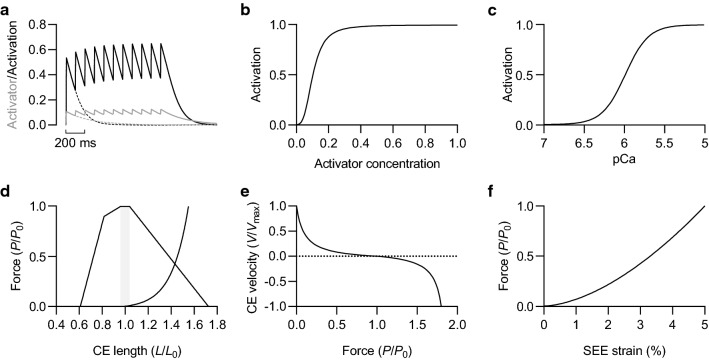
Muscle model properties. **a** Activator concentration (lower trace) and activation level during a twitch (dashed) and 1 s stimulation train at 10 Hz. **b** Activation-activator relationship. **c** Activation-pCa relationship. **d** CE and PEE force–length relationships. Shaded region represents range of optimal CE lengths. **e** CE force–velocity relationship. **f** SEE force-strain relationship

Activation of the CE is regulated by the concentration of an activator, which we consider to be calcium, in a single compartment. Calcium ions (Ca^2+^) are released transiently at a constant rate in response to each stimulus and subsequently removed at a rate dependent on the Ca^2+^ concentration (Fig. 1a). Ca^2+^ release occurs over a defined pulse width according to the following equation:

$$\frac{da}{dt}= \frac{(1-a)}{{\tau }_{1}}$$

Otherwise, Ca^2+^ is removed according to the following equation:

$$=\frac{-a}{{\tau }_{2}}$$

where *a* is the concentration of activator (Ca^2+^) and *τ*_1_ and *τ*_2_ are the time constants for the rise and fall of Ca^2+^, respectively (Lichtwark and Wilson 2005a). Ca^2+^ release for a second stimulus is attenuated relative to the first for brief interstimulus intervals (Caputo et al. 2004; Barclay 2012). Inactivation of Ca^2+^ release was incorporated into the model by reducing pulse width according to a single exponential equation describing the recovery of Ca^2+^ release with respect to interstimulus interval:

$$1-A{e}^{-isi/r}$$

where *A* is the minimum relative Ca^2+^ release (i.e. maximum inactivation), *isi* is the interstimulus interval and *r* is the time constant for the recovery of relative Ca^2+^ release (Barclay 2012). *A* and *r* were set at 20% and 350 ms, respectively, such that the force-frequency relationship was comparable to empirical observations for predominantly slow muscle [e.g. rat soleus (Ranatunga 1982; Larsson and Edström 1986)] or several muscles with varying fibre type compositions crossing the same joint (Marsh et al. 1981; Sale et al. 1982).

The relationship between Ca^2+^ and activation (Fig. 1b) is given by a sigmoidal function of the form:

$$Act= \frac{{a}^{{n}_{H}}}{({a}^{{n}_{H}}+{{a}_{50}}^{{n}_{H}})}$$

where *Act* is thin filament activation and represents the fraction of cross-bridge binding sites available for cycling, *n*_H_ is the Hill coefficient, and *a*_50_ is the activator concentration required for half-maximal cross-bridge activation (Curtin et al. 1998). *n*_H_ and *a*_50_ (i.e. pCa_50_) are indices of cooperative activation and the Ca^2+^ sensitivity of force, respectively (Walker et al. 2010).

To relate the activation-activator relationship in the model to the force-pCa relationship of permeabilised single fibres (Hellam and Podolsky 1969; Stephenson and Williams 1982), we assumed that the activator concentration achieving saturation was equivalent to a calcium concentration of 10 μM or pCa 5 (Fig. 1c); pCa is the negative log of the theoretical calcium concentration. A form of the Hill equation was then used to describe the relationship between calcium concentration and force:

$$\frac{P}{{P}_{0}}=\frac{1}{1+{10}^{nH}\cdot ({\mathrm{pCa}-\mathrm{pCa}}_{50})}$$

where pCa is the negative log of the activator concentration, and pCa_50_ is the negative log equivalent of *a*_50_ (Martyn and Gordon 2001).

#### CE force–length & force–velocity relationships

Active force generated by the CE was modelled according to classic force–length (Gordon et al. 1966) and force–velocity (Hill 1938) relationships (Fig. 1d, e). The speed at which the CE shortens with respect to force, which is scaled by CE activation and length, was modelled according to a normalised form of the Hill equation:

$$\frac{P}{{P}_{0}}=\frac{K\left(1-V/{V}_{\mathrm{max}}\right)}{K+V/{V}_{\mathrm{max}}}$$

where *P* is force, *P*_0_ is the maximum isometric force, *K* represents a/*P*_0_ and indicates the curvature of the rectangular hyperbola describing the concentric force–velocity relationship, *V* is the velocity of CE shortening and *V*_max_ is the maximum velocity of CE shortening (Seow 2013). The speed at which the CE lengthens with respect to an externally applied force (Fig. 1e) was modelled according to the following equation:

$$\frac{P}{{P}_{0}} =c-\frac{k\left(1+V/{V}_{\mathrm{max}} \right)}{1-q(V/{V}_{\mathrm{max}})}$$

where *c* is a constant indicating the maximum eccentric force expressed relative to *P*_0_, *k* is a constant relating to the *y*-intercept, whereby $c-k=1$ to meet the condition of $P/{P}_{0}=1$ for a lengthening velocity of 0, and *q* is a constant describing the curvature of the eccentric force–velocity relationship (Otten 1987; Azizi and Roberts 2014). *c*, *k,* and *q* were assigned values of 1.8, 0.8 and 7, respectively.

#### PEE force–length relationship

The PEE was assigned an exponential passive force–length relationship (Fig. 1d) according to the following exponential equation:

$$\frac{P}{{P}_{0}}=\frac{{e}^{{k}_{\mathrm{PEE}}({L}_{\mathrm{CE}}-{L}_{\mathrm{slack}})/{\varepsilon }_{\mathrm{PEE}}}-1}{{e}^{{k}_{\mathrm{PEE}}}-1}$$

where *k*_PEE_ is an exponential shape factor, *L*_CE_ is the relative CE length, *L*_slack_ is the slack length of the PEE, and *ε*_PEE_ is the passive strain of the CE when an external load equal to *P*_0_ is applied (Thelen 2003). When *L*_slack_ is not equal to 1.0, the CE length at *ε*_PEE_ is equal to *L*_slack_ plus *ε*_PEE_. *k*_PEE_, *L*_slack_ and *ε*_PEE_ were assigned values of 4, 0.98 and 0.57, respectively. These values are similar to those used previously for human plantar flexors (Thelen 2003) and generate a passive-force length relationship generally consistent with experimental data (Winters et al. 2011; Rubenson et al. 2012; Moo et al. 2020).

#### SEE force–length relationship

The SEE was assigned a non-linear load-deformation relationship consistent with experimental data for tendon and aponeurosis (Lieber et al. 1991; Trestik and Lieber 1993; Zuurbier et al. 1994; Loren and Lieber 1995; Cui et al. 2009). The general relationship was derived from a non-linear least squares fit of force and deformation data reported for mammalian tendon (Bennett et al. 1986) using the following equation:

$$P=a{x}^{b}$$

where *a* and *b* are regression constants and *x* is tendon deformation. SEE strain at *P*_0_ (*ε*_SEE_, Fig. 1f) was set to 0.05 (Muramatsu et al. 2001; Arampatzis et al. 2005; Karamanidis and Arampatzis 2006). SEE stiffness was defined as the maximum deformation of the SEE normalised to *P*_0_ and the optimum length of the CE (*L*_0_), giving a normalised stiffness [*k*_SEE_ (Lichtwark and Wilson 2005b)]. SEE length (*L*_SEE_) and *L*_0_ were set at 300 (Arampatzis et al. 2005; Karamanidis and Arampatzis 2005) and 50 mm (see Hessel et al. 2021 main text and supplementary data), respectively, giving a *k*_SEE_ of 3.33 *P*_0_·*L*_0_^−1^. The inverse of normalised SEE stiffness—normalised SEE compliance (i.e. 30%)—relates closely to the fixed-end compliance, which represents CE strain against the stretch of the SEE during a maximum tetanic contraction (Roberts 2002). We have instead defined fixed-end compliance as CE shortening expressed relative to *L*_0_, as to allow normalised SEE compliance and fixed-end compliance to be equal. Because there is considerable passive tension at the optimal MTU length in the model (initial CE length of ~ 1.23 *L*_0_), consistent with experimental observations for the human plantar flexors (see Hessel et al. 2021 supplementary data), SEE deformation and CE shortening during maximum force development are ~ 23%, rather than 30% of *L*_0_. This value generally agrees with experimental data for the human plantar flexors (see Hessel et al. 2021 supplementary data) after considering the overestimation of fascicle shortening against the stretch of tendon and aponeurosis owing to inevitable ankle rotation (Karamanidis et al. 2005).

### Model optimisation to simulate plantar flexion twitch of young men

Model parameters for the initial state or control condition were optimised to minimise the combined error in contraction time and half-relaxation time between simulated and experimental twitches. An additional requirement was that the relative amplitude of the simulated twitch be ~ 0.2 *P*_0_. The simulated twitch was for an initial CE length of 1.0 *L*_0_. The experimental twitch represented the waveform average for young men determined from twitches recorded with the ankle at 0° and the knee extended. Initially, *τ*_1_, *τ*_2_, [a]_50_, *n*_H_ and *V*_max_ parameters were included in the optimization process. Where appropriate, physiological upper and lower limits were imposed. Subsequently, *n*_H_ and *V*_max_ were constrained at values of 3 and 6, respectively, and the optimization process was repeated.

Although reported values of *n*_H_ are wide-ranging, a value of 3 is generally intermediate between values reported for type I and II fibres or similar to values reported for the former (Stephenson and Williams 1981; Fink et al. 1986, 1990; Lynch et al. 1991; Hvid et al. 2011, 2013). The *V*_max_ value of 6 *L*_0_·s^−1^ agrees closely with the value of 6.2 FL·s^−1^ (fibre lengths per second) measured for human medial gastrocnemius fascicles in vivo (Hauraix et al. 2015), and is comparable to values reported for rat soleus [6–7.3 FL·s^−1^ (Ranatunga and Thomas 1990; Ranatunga 1998)] and mouse soleus [4.5 and 8.6 FL·s^−1^ (Luff 1981; Lichtwark and Barclay 2010)] muscles at physiological temperatures; the type I fibre composition of these muscles is approximately 73 and 67%, respectively (Asmussen and Maréchal 1989). We arrived at a slightly lower estimate of 60% for the MHC I fibre content for the triceps surae [see section ‘2.3.10 Type I fibre fractional area (i.e., MHC I fibre content)’].

### Simulating ageing-related adaptations

#### Free Ca^2+^ concentration

There is evidence that both SR Ca^2+^ release and SR Ca^2+^ uptake are impaired in advanced age (Larsson and Salviati 1989; Delbono et al. 1995; Narayanan et al. 1996; Hunter et al. 1999; Wang et al. 2000; Jiménez-Moreno et al. 2008), and that aged single fibres can exhibit a deficit in peak free Ca^2+^ concentration (González et al. 2003; Andersson et al. 2011; Umanskaya et al. 2014). To our knowledge, the effect of slowed SR Ca^2+^ uptake on steady-state free Ca^2+^ concentration and free Ca^2+^ decay in intact fibres has not been studied in advanced age. It is unclear whether both adaptations can coexist (Russ et al. 2011, 2014) and to what extent each alteration influences free Ca^2+^ concentration. Accordingly, we simulated the independent and concomitant effects of impaired Ca^2+^ release and slowed Ca^2+^ uptake. For simplicity, we assume that the reductions in peak free Ca^2+^ concentration reported in the literature reflect impaired Ca^2+^ release without a concomitant slowing of SR Ca^2+^ uptake. This simplification allows experimental values of the deficit in Ca^2+^ concentration in intact fibres to be emulated by scaling down the instantaneous Ca^2+^ availability in the model, rather than increasing the time constant of Ca^2+^ release, *τ*_1_. Importantly, the fractional deficit in peak free Ca^2+^ concentration in intact fibres associated with ageing appears to be similar for maximal and submaximal contractions (González et al. 2003; Eshima et al. 2020).

To our knowledge, there exists only one study of the effect of age on SR Ca^2+^ release in human single fibres (Delbono et al. 1995), whereas several studies have been performed on rodent single fibres. In these studies, intact fibres were isolated exclusively from fast-twitch muscles without fibre type identification (Wang et al. 2000, 2002; González et al. 2003; Jiménez-Moreno et al. 2008; Andersson et al. 2011; Umanskaya et al. 2014; Fodor et al. 2020; Eshima et al. 2020). Each of these studies, including the study on human type II fibres, demonstrated an age-related deficit in SR Ca^2+^ release rate or peak intracellular Ca^2+^ concentration. Collectively, impairment typically ranged from ~ 30–50%. Accordingly, we incorporated a 30 or 50% reduction in peak Ca^2+^ concentration by applying a scaling factor to the instantaneous Ca^2+^ concentration of 0.7 or 0.5, respectively. We described the qualitative and quantitative effect (% change) of lower free Ca^2+^ availability on the time course (i.e., contraction time, half-relaxation time) and amplitude of the twitch, submaximal force and the force-frequency relationship, and maximum force.

#### SR Ca^2+^ uptake (*τ*_2_)

The rate of SR Ca^2+^ uptake may be lower in advanced age, but it is not a universal observation. The effect of age on SR Ca^2+^ uptake rate has been predominantly studied in rodent muscle and using a variety of muscle preparations. To our knowledge, only a single study has been performed on human muscle, specifically, crude homogenates form the vastus lateralis muscle (Hunter et al. 1999). In many regards, the findings are inconsistent. Slowing has been demonstrated for skinned type II fibres but not skinned type I fibres (Larsson and Salviati 1989), SR vesicles isolated from slow-twitch muscle but not fast-twitch muscle (Narayanan et al. 1996; Russ et al. 2014), and muscle homogenates from fast-twitch (Russ et al. 2011), slow-twitch (Narayanan et al. 1996), and mixed-fibre type (Hunter et al. 1999) muscles but not in every instance (Fitts et al. 1984; Narayanan et al. 1996; Thomas et al. 2010); measurements from muscle homogenates may be susceptible to confoundment by a shift in MHC isoform composition. For those studies supporting an age-related reduction in Ca^2+^ uptake rate, the size of the slowing effect ranged from ~ 20–52% (Larsson and Salviati 1989; Narayanan et al. 1996; Hunter et al. 1999; Russ et al. 2011). Accordingly, we incorporated a 30 or 50% reduction in the rate constant for Ca^2+^ removal. The rate constant for Ca^2+^ removal is the reciprocal of the time constant, *τ*_2_. Therefore, *τ*_2_ was increased by 43 and 100%. We described the qualitative and quantitative effect of slower Ca^2+^ uptake on the time course and amplitude of the twitch, and submaximal force and the force-frequency relationship.

#### Ca^2+^ sensitivity (pCa_50_)

Lower Ca^2+^ sensitivity of force has been found in advanced age, but it is not a universal observation. Studies showing no effect of age (Plant and Lynch 2001; Hvid et al. 2011, 2013, 2017; Lamboley et al. 2015; Teigen et al. 2020; Mazara et al. 2021) are similar in number to those showing an age-related deficit. Reduced Ca^2+^ sensitivity in advanced age has been demonstrated by several studies for type II fibres from human (Lamboley et al. 2015; Straight et al. 2018; Mazara et al. 2021) and rodent (Brooks and Faulkner 1994; Lowe et al. 2002) muscles. In contrast, only a single study has shown Ca^2+^ sensitivity to be lower for type I fibres (Straight et al. 2018). The deficit in pCa_50_ reported by these studies ranges from 0.05 to 0.15 pCa units but is typically ~ 0.10 pCa units. Three studies reported a similar difference between means (0.08–0.10 pCa units) without detecting a significant age effect (Hvid et al. 2011, 2013; Mazara et al. 2021). For two of those studies, which sampled from just 11–15 aged type II fibres, we could deduce that the deficit in pCa_50_ was of a moderate effect size (Cohen's *d* = 0.38–0.49). Accordingly, we incorporated a reduction in pCa_50_ of 0.05 or 0.10 pCa units and described the qualitative and quantitative effect on the time course and amplitude of a twitch, submaximal force and the force-frequency relationship, maximum force, and relative force summation.

#### Cooperativity of activation (***n***_H_)

The weight of evidence from human and rodent studies indicates that the slope of the force-pCa relationship (i.e. cooperativity)—represented by the Hill coefficient, *n*_H_—is unaltered in advanced age (Eddinger et al. 1986; Brooks and Faulkner 1994; Hvid et al. 2011, 2013; Lamboley et al. 2015; Straight et al. 2018; Teigen et al. 2020). Challenging this view is one study on human muscle that found *n*_H_ to be elevated for type II fibres in advanced age (Straight et al. 2018) and one study on rat muscle that found *n*_H_ (> 0.5 *P*_0_) to be lower for type II fibres (Lowe et al. 2002). Because there are generally pronounced differences in *n*_H_ between fibre types (Fink et al. 1986, 1990; Gardetto et al. 1989; Danieli-Betto et al. 1990; Gregorevic et al. 2004; Hvid et al. 2011, 2013), and stronger evidence that *n*_H_ is affected by disuse (Gardetto et al. 1989; Widrick et al. 1998; Hvid et al. 2011, 2013; Monti et al. 2021), we thought it was important to illustrate the effect of this parameter on force generation. We performed simulations in which the reference value of *n*_H_ was increased and decreased by 1.0. We described the qualitative and quantitative effect on the time course and amplitude of a twitch, submaximal force and the force-frequency relationship, and relative force summation.

#### Lower free Ca^2+^ concentration & lower Ca^2+^ sensitivity (pCa_50_) in concert

Because the force-pCa relationship is sigmoidal in form, force at near-maximal Ca^2+^ concentrations is practically insensitive to shifts in Ca^2+^ sensitivity. A deficit in maximum force generation may only arise when Ca^2+^ concentration and Ca^2+^ sensitivity decrease concomitantly. Accordingly, we reduced the instantaneous Ca^2+^ concentration by 30 or 50% whilst lowering pCa_50_ by 0.05 or 0.10 pCa units, consistent with our previous manipulations of Ca^2+^ concentration and Ca^2+^ sensitivity. We described the qualitative effect of concurrent reductions in Ca^2+^ concentration and Ca^2+^ sensitivity on submaximal force and the force-frequency relationship, and quantified the effect on maximal force.

#### Lower Ca^2+^ release & slower Ca^2+^ uptake (*τ*_2_) in concert

It is unclear from recordings of intracellular Ca^2+^ transients whether impaired Ca^2+^ release and slowed Ca^2+^ uptake occur in parallel and the extent to which they may offset one another (González et al. 2003; Andersson et al. 2011; Eshima et al. 2020). Accordingly, we reduced Ca^2+^ release by 30% and the rate constant of Ca^2+^ uptake by 40 (67% increase in *τ*_2_) in concert. Reductions of equal amount would not alter steady-state Ca^2+^ in the model relative to the control condition. We described the qualitative effect of lower Ca^2+^ release and slower Ca^2+^ uptake on submaximal force and the force-frequency relationship.

#### Lower Ca^2+^ sensitivity (pCa_50_) and slower Ca^2+^ uptake (*τ*_2_) in concert

On the basis that slower Ca^2+^ uptake will increase free Ca^2+^ concentration, thereby increasing force, whereas lower Ca^2+^ sensitivity will act to decrease force, we examined the effect of these two adaptations acting concomitantly. Specifically, we incorporated both a 30% reduction in the rate constant for Ca^2+^ removal (43% increase in *τ*_2_) and a 0.1 pCa unit reduction in Ca^2+^ sensitivity (pCa_50_) and described the qualitative effect on the force-frequency relationship.

#### Maximum velocity of shortening (***V***_max_)

Comparatively, the intrinsic speed of shortening has been extensively studied in young and old muscle fibres from humans and rodents. For both groups, and for both fibre types, the effect of age is inconsistent. Several studies have demonstrated slowing of either or both type I and type II human fibres (Krivickas et al. 2001; D’Antona et al. 2003; Ochala et al. 2007; Yu et al. 2007; Power et al. 2016; Brocca et al. 2017), but just as many studies have found no age effect (Trappe et al. 2003; Claflin et al. 2011; Sundberg et al. 2018; Teigen et al. 2020; Grosicki et al. 2021; Mazara et al. 2021). Similarly, a lower maximal shortening velocity has been demonstrated for aged type I and type II fibres from rodents (Degens et al. 1998; Thompson and Brown 1999; Kim and Thompson 2013), but not in every instance (Eddinger et al. 1986; Brooks and Faulkner 1994; Zhong et al. 2006; Kim and Thompson 2013). Muscle inactivity in advanced age may minimize or negate the effect of age on *V*_max_ (Thompson et al. 1998; D’Antona et al. 2003; Kim and Thompson 2013), although there is evidence to the contrary (Grosicki et al. 2021). The deficit in maximal shortening velocity reported for human muscle fibres ranges from ~ 7–46% (Larsson et al. 1997; Krivickas et al. 2001; Claflin et al. 2011; Power et al. 2016) but is most often ~ 15–25% (Larsson et al. 1997; Krivickas et al. 2001; D’Antona et al. 2003; Ochala et al. 2007; Yu et al. 2007; Brocca et al. 2017). The slowing of intrinsic shortening speed tends to be more pronounced for rodent muscle fibres; three of the four identified instances of slowing represent a deficit of 32–50% (Degens et al. 1998; Thompson and Brown 1999; Kim and Thompson 2013). Accordingly, we incorporated reductions in intrinsic shortening speed of 30 or 50% and described the qualitative and quantitative effect on the time course and amplitude of a twitch and brief tetanic contraction.

The curvature of the force–velocity relationship (i.e. a/*P*_0_) does not appear to be altered in aged fibres that do not exhibit a reduction in *V*_max_ (Brooks and Faulkner 1994; Trappe et al. 2003). To our knowledge, a/*P*_0_ has not been quantified for aged fibres exhibiting a reduction in *V*_max_. However, because a/*P*_0_ differs markedly between fibre types—human type IIa fibres compared to type I fibres exhibit a two-fold greater value of a/*P*_0_—(Bottinelli et al. 1996; Widrick et al. 1996; Gilliver et al. 2009)—we deemed it important to illustrate the effect of this parameter on force development. We performed simulations in which the reference value of a/*P*_0_ was increased by 0.05 (50%). We described the qualitative effect on the time course and amplitude of a twitch and brief tetanic contraction. We did not incorporate reductions in a/*P*_0_ because the model was unable to simulate the twitch measured experimentally with a more realistic value for muscle at physiological temperatures [model: 0.10; mouse soleus: 0.18 (Luff 1981); rat soleus: 0.22–0.26 (Ranatunga and Thomas 1990; Ranatunga 1998)].

#### SEE stiffness

The general effect of age on tendon stiffness appears to be distinct for humans compared to certain animal models of ageing. The weight of evidence from in vivo human studies favours an age-associated reduction in tendon stiffness and elastic modulus (see McCrum et al., 2018). Though some studies have found tendon loading behaviour to be unchanged (Carroll et al. 2008; Couppé et al. 2012), no human studies appear to have reported an age-related increase in tendon stiffness. A recent review reported median reductions in stiffness and elastic modulus of 20% and 28%, respectively (McCrum et al. 2018). However, age-related deficits may be as high as 30–55% (Karamanidis and Arampatzis 2006; Onambele et al. 2006; Stenroth et al. 2012; Csapo et al. 2014). Moreover, relative to old adults (> 65 years), very old adults (> 83 years) can exhibit marked reductions (35–40%) in tendon stiffness and elastic modulus (Eriksen et al. 2018).

In contrast to humans, hindlimb tendons of rodents in advanced age regularly exhibit higher stiffness or elastic modulus (Wood et al. 2011; Danos et al. 2016; Wood and Brooks 2016; Leahy et al. 2022). The magnitude of the increase is typically close to 50%. A number of studies have also reported no ageing effect (Nakagawa et al. 1996; Pardes et al. 2017); fewer have reported an age-related reduction (LaCroix et al. 2013). The variable nature of tendon mechanics in advanced age may be partially explained by methodological approach, age at measurement, ageing-associated inactivity, species, and muscle function (Svensson et al. 2016; McCrum et al. 2018).

We incorporated both a reduction in normalised SEE stiffness of 30% and an increase in normalised stiffness of 50%. We described the qualitative and quantitative effect of SEE stiffness on the time course and amplitude of a twitch and brief tetanic contraction.

#### Type I fibre fractional area (i.e., MHC I fibre content)

Several human and rodent studies have reported an ageing-related increase of 0.10–0.20, or greater (Brocca et al. 2017), for fractional MHC I content or fractional area occupied by type I fibres (Larsson et al. 1978; Klitgaard et al. 1990b; Kadhiresan et al. 1996; Short et al. 2005; Cui et al. 2008; Nilwik et al. 2013; Sonjak et al. 2019; Soendenbroe et al. 2022). Smaller shifts have also been reported (Sullivan et al. 1995; Hunter et al. 1999). We approximated the effect of this adaptation by considering fibre type-related differences in Ca^2+^ removal rate, Ca^2+^ sensitivity and cooperativity, and *V*_max_, and by adjusting composite parameter values according to an increase in type I fibre fractional area. Fibre type-specific values and composite relationships were derived from the whole muscle control values by assigning weightings based on the fractional cross-sectional area of type I and II fibres and assigning fibre type-related differences in each property (Wakeling et al. 2012). Fibre type-specific and composite parameter values were determined according to the following expression:

$$({x}_{\mathrm{slow}}\times {p}_{\mathrm{slow}})+({x}_{\mathrm{fast}}\times {p}_{\mathrm{fast}})= {x}_{\mathrm{whole}}, \frac{{x}_{\mathrm{slow}}}{{x}_{\mathrm{fast}}}=y$$

where *x* is the fibre type-specific parameter value, *p* is relative fibre content, *x*_whole_ is the whole muscle parameter value, and *y* is the fibre type-related difference or offset. Some empirical observations support this approach. Single fast and slow fibres arranged in parallel exhibit an intermediate force-pCa relationship generally consistent with a theoretical composite relationship based on fractional fibre type content (Lynch et al. 1995). In contrast to some methods (Zajac 1989; Claflin and Faulkner 1989; Ranatunga and Thomas 1990), our approach assumes some degree of attenuation of shortening speed at loads where whole muscle velocity exceeds the *V*_max_ assigned to type I fibres, which is generally consistent with observations that inactive muscle depresses the speed of shortening (Hatcher and Luff 1987; Holt et al. 2014).

The total MHC isoform content or fractional area occupied by type I fibres is approximately 50 and 65% for the gastrocnemius and soleus muscles, respectively (Edström and Nyström 1969; Green et al. 1981; Coggan et al. 1992; Harridge et al. 1996, 1998). Thus, given that the combined physiological cross-sectional area (PCSA) of the lateral and medial gastrocnemius muscles represents ~ 38% of the total PCSA of the triceps surae (Morse et al. 2005; Albracht et al. 2008; Crouzier et al. 2018), it is estimated that the total type I fibre area of the triceps surae is ~ 60% (i.e. 0.60). Type I fibres, compared to type II fibres, were assumed to exhibit the following differences: Ca^2+^ removal rate constant 50% slower (Carroll et al. 1997; Liu et al. 1997; Baylor and Hollingworth 2003; Calderón et al. 2010); Ca^2+^ sensitivity (pCa_50_) 0.15 pCa units greater and *n*_H_ 40% lower (Stephenson and Williams 1981; Fink et al. 1986, 1990; Ruff 1989; Laszewski-Williams et al. 1989; Gardetto et al. 1989; Ruff and Whittlesey 1991; Plant and Lynch 2001; Gregorevic et al. 2004; Hvid et al. 2013; Xu et al. 2017; Lamboley et al. 2020); *V*_max_ 70% slower (Larsson and Moss 1993; Bottinelli et al. 1996; Harridge et al. 1996; Widrick et al. 2002; Trappe et al. 2003; Yu et al. 2007; Luden et al. 2008; Sundberg et al. 2018; Teigen et al. 2020). Note that much variability exists for the force-pCa relationships of type I and type II fibres.

After increasing the fractional area of type I fibres by 0.10 and 0.20, from 0.60 to 0.70 and 0.80, respectively, and incorporating adjusted values of *τ*_2_, pCa_50_, *n*_H_ and *V*_max_, we described the qualitative and quantitative effect on the contraction time and half-relaxation time of the twitch, and on submaximal force and the force-frequency relationship.

### Model optimisation to simulate plantar flexion twitch of old men

The parameters of the model were adjusted to simulate the plantar flexion twitch of old men. The optimisation approach was similar to that used initially, except parameter limits were imposed consistent with the directionality of impairment. We also incorporated a force-generating capacity (FGC) parameter—analogous to muscle PCSA—to allow twitch force to be lower in advanced age, which we observed experimentally. We assumed that the deficit in twitch force is owing, at least in part, to muscle atrophy (Narici et al. 2003; Morse et al. 2005; Thom et al. 2007). The magnitude of a given parameter adjustment was compared to the parameter change incorporated to simulate age-related adaptation reported in the literature.

## Results

### Model optimisation to simulate plantar flexion twitch of young men

**Fig. 2:**
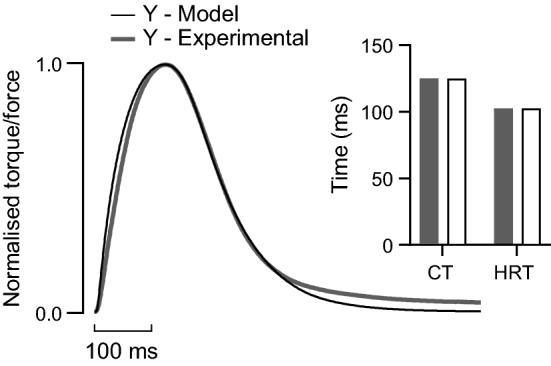
Hill-type muscle model optimisation. Experimental plantar flexion twitch torque and simulated twitch force for young (Y) adult humans. *Inset*, twitch contraction time (CT) and half-relaxation time (HRT) for experimental (filled) and simulated (open) data were identical following parameter optimization (CT: 125 ms; HRT: 102.5 ms). Agreement was achieved between twitch torque measured with the ankle at 0° and the knee extended and twitch force simulated at an initial CE length of 1.0 *L*_0_

Force during simulated and experimental twitches are shown in Fig. 2. The time course of force rise and decay are matched well, with both the contraction time and half-relaxation time of the simulated twitch being identical to the values measured experimentally for young men (*inset* Fig. 2). All model parameters used in simulations of young muscle (i.e., control condition) are reported in Table 1. Unless specified otherwise, all simulated fixed-end contractions were performed at an initial CE length of 1.23 *L*_0_ and force displayed in figures represents active CE force. This initial length for a maximal tetanic contraction resulted in a final CE length—after shortening against the stretch of the SEE—on the plateau of the force–length relationship.

**Table 1:** Model parameters for plantar flexors muscles of young adult men

Pulse width	*τ*_1_	*τ*_2_	[a]_50_	pCa_50_	*n*_H_	*V*_max_	a/*P*_0_	*ε*_SEE_	*k*_SEE_	*L*_0_	*L*_SEE_
(s)	(s)	(s)		(pCa)		(*L*_0_·s^−1^)			(*P*_0_·*L*_0_^−1^)	(mm)	(mm)
0.0048	0.0422	0.256	0.1025	5.99	3	6	0.10	0.05	3.33	50	300

### Ca^2+^ handling and thin filament activation

#### Instantaneous Ca^2+^ concentration

**Fig. 3:**
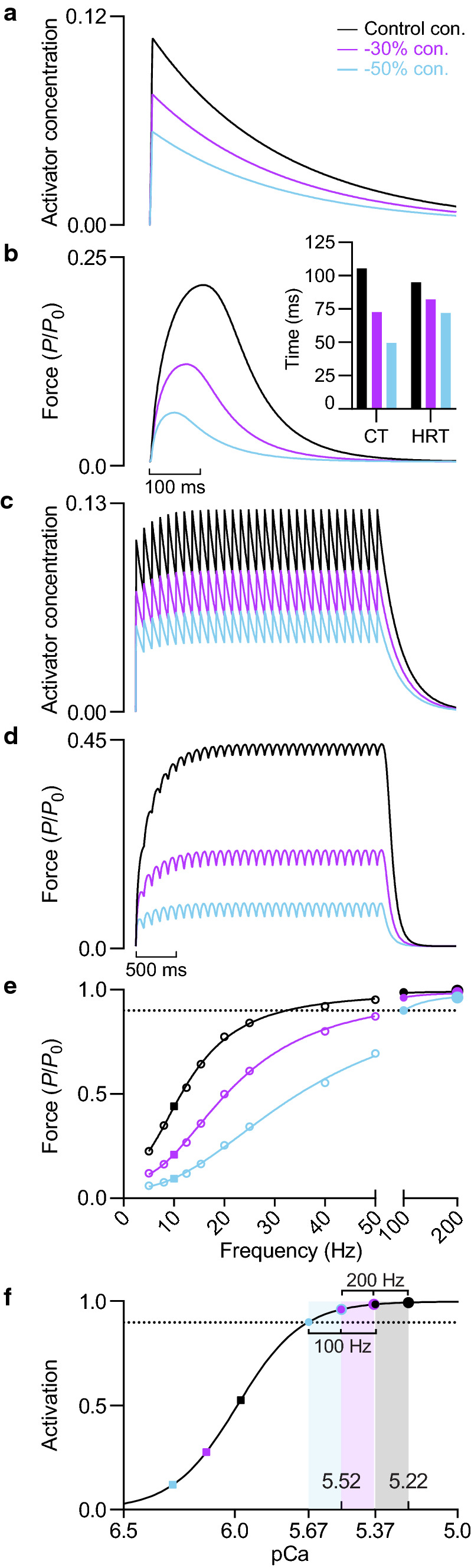
Effect of calcium concentration on submaximal and maximal force generation. **a** Activator concentration and **b** active force during a twitch at 1.23 *L*_0_ with varying levels of instantaneous Ca^2+^ concentration (con.). Instantaneous Ca^2+^ concentration was reduced by 30 and 50% relative to the control condition. *Inset*, twitch contraction time and half-relaxation time. **c** Activator concentration and **d** active force during sustained 10 Hz stimulation at 1.23 *L*_0_. ***e*** Force-frequency relationships at 1.23 *L*_0_. Active force expressed relative to *P*_0_ of control condition. **f** Activation-pCa relationships. In *e* and *f*, solid squares denote 10 Hz stimulation and small and large solid circles denote 100 and 200 Hz stimulation, respectively. In *f*, shaded regions represent the Ca^2+^ concentration range between 100 and 200 Hz

Reducing the instantaneous free Ca^2+^ concentration by either 30 or 50% dramatically lowered twitch force and abbreviated the rise and decay of twitch force (Fig. 3b, *inset*). Substantial force loss was also evident during sustained stimulation at submaximal frequencies, as shown by the marked rightward shift of the force-frequency relationship (Fig. 3d, e). There was a pronounced deficit in force for frequencies yielding calcium concentrations situated on the steep region of the force-pCa relationship (Fig. 3e, f). In contrast, maximum tetanic force was only modestly affected by the imposed reductions in free Ca^2+^ concentration (Fig. 3e, f). During 200 Hz stimulation, the 50% reduction in Ca^2+^ availability only lowered cross-bridge activation to 97%. Because submaximal force was disproportionately affected, reduced Ca^2+^ availability lowered the ratio of twitch-to-tetanic force.

#### Ca^2+^ uptake

**Fig. 4:**
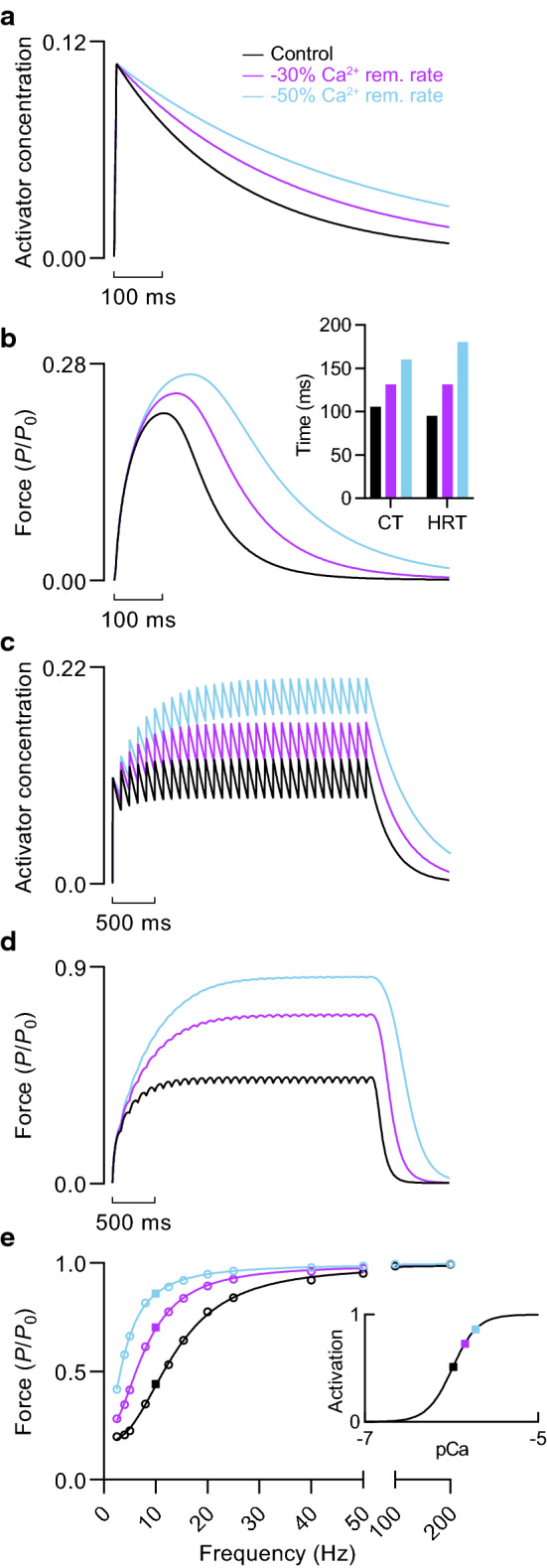
Effect of calcium uptake rate on force generation at submaximal calcium. **a** Activator concentration and **b** active force during a twitch at 1.23 *L*_0_ with varying rates of Ca^2+^ removal (rem.). The rate constant of Ca^2+^ removal was reduced by 30 and 50% relative to the control condition, representing increases in the time constant of Ca^2+^ removal, *τ*_2_, of 43 and 100%, respectively. *Inset*, twitch contraction time and half-relaxation time. **c** Activator concentration and **d** active force during sustained 10 Hz stimulation at 1.23 *L*_0_. **e** Force-frequency relationships at 1.23 *L*_0_. Active force expressed relative to *P*_0_ of control condition. *Inset*, activation-pCa relationship. Solid squares denote the activator concentration and activation level during sustained stimulation at 10 Hz

Decreasing the rate constant of Ca^2+^ decay by 30% to slow the removal of Ca^2+^ increased twitch force by 12% (elevating the ratio of twitch-to-tetanic force) and prolonged the contraction time and half-relaxation time by 25% and 38%, respectively (Fig. 4b, inset). Slowing Ca^2+^ uptake by 50% increased the size of these effects. Slower Ca^2+^ removal during sustained stimulation resulted in greater calcium accumulation (Fig. 4c), which caused force at submaximal frequencies to increase dramatically (Fig. 4d), as illustrated by the leftward-shifted force-frequency relationships (Fig. 4e).

#### Calcium sensitivity (pCa_50_)

**Fig. 5:**
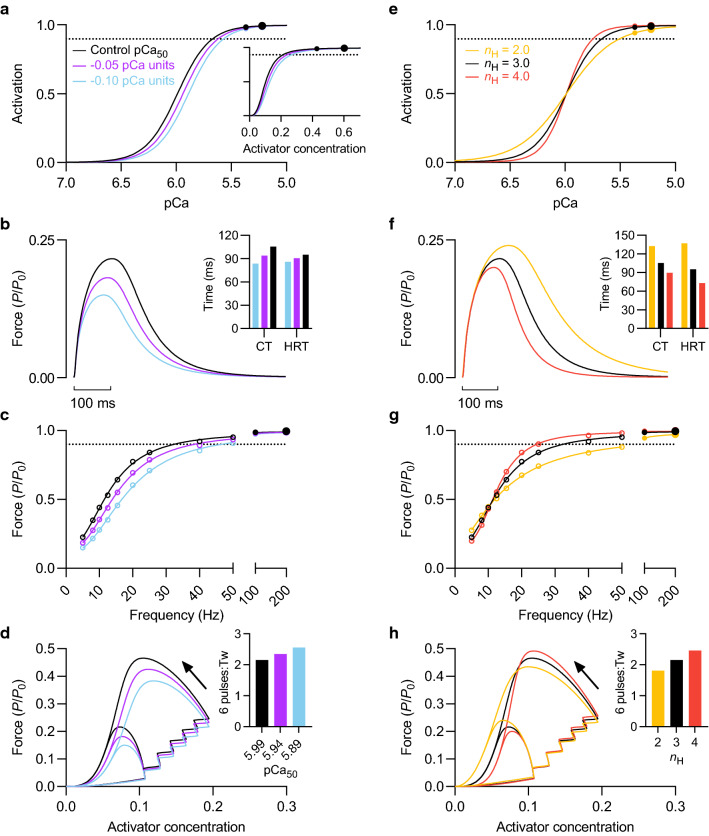
Effect of calcium sensitivity and cooperativity on force at submaximal and maximal calcium concentrations. **a** Activation-pCa relationships with varying Ca^2+^ sensitivities and **e** cooperativities. Ca^2+^ sensitivity of force was reduced by 0.05 and 0.10 pCa units relative to the control condition. Cooperativity was decreased from 3 to 2 and increased to 4. **a** *Inset*, corresponding activation-activator relationships. Small and large black circles denote 100 and 200 Hz stimulation, respectively. **b***, ***f** Twitch force at 1.23 *L*_0_. *Inset*, twitch contraction time and half-relaxation time. **c***, ***g** Force-frequency relationships at 1.23 *L*_0_. Active force expressed relative to *P*_0_ of control condition. **d***, ***h** Active force plotted as a function of activator concentration for a 50 ms, 100 Hz train (i.e., 6 pulses) at 1.23 *L*_0_. *Inset*, force summation; the ratio of tetanic (50 ms, 100 Hz) force-to-twitch force

Reducing the Ca^2+^ sensitivity of force by shifting the force-pCa relationship rightward 0.05 pCa units reduced twitch force by 16% and abbreviated the twitch contraction time and half-relaxation time by 11 and 5%, respectively (Fig. 5b, *inset*). Lowering Ca^2+^ sensitivity by 0.10 pCa units resulted in additional force attenuation and an even briefer contraction. Reduced Ca^2+^ sensitivity also decreased force during stimulation at submaximal stimulation frequencies (< 50 Hz) such that the force-frequency relationship was shifted rightward (Fig. 5c). Force loss was greatest for frequencies that encompassed the steep region of the force-pCa relationship and increased in proportion to the reduction in Ca^2+^ sensitivity. Maximum tetanic force was unaffected by the imposed reductions in Ca^2+^ sensitivity; no force loss was evident for 100 or 200 Hz stimulation. Relative force summation—illustrated as the force during a brief tetanic contraction (50 ms, 100 Hz) expressed relative to twitch force (Fig. 5d, *inset*)—was higher following the reduction in Ca^2+^ sensitivity, though there remained a deficit in tetanic force.

#### Cooperativity of activation (***n***_H_)

Decreasing *n*_H_, or cooperativity, from 3 to 2 to reduce the slope of the force-pCa relationship increased twitch force by 11% and prolonged the twitch contraction time and half-relaxation time by 26 and 44%, respectively (Fig. 5f, *inset*). Lowering cooperativity also reduced the slope of the force-frequency relationship such that force was slightly greater at low frequencies but considerably lower at moderate and high frequencies of stimulation (Fig. 5g); increasing cooperativity had the opposite effect. Again, maximum tetanic force was largely unaffected by altering cooperativity. A small deficit (2%) in maximum cross-bridge activation level arose when cooperativity was lowered. As such, reducing cooperativity increased the ratio of twitch-to-tetanic force, whereas increasing cooperativity reduced this ratio. Similarly, lowering cooperativity also reduced relative force summation (Fig. 5h, *inset*). Despite twitch force being 20% (0.04 *P*_0_) greater for a *n*_H_ of 2 compared to a *n*_H_ of 4, peak force during the brief tetanic contraction (50 ms, 100 Hz) was 12% (0.06 *P*_0_) lower for the former compared to the latter (Fig. 5h).

#### Lower Ca^2+^ concentration & lower Ca^2+^ sensitivity in concert

**Fig. 6:**
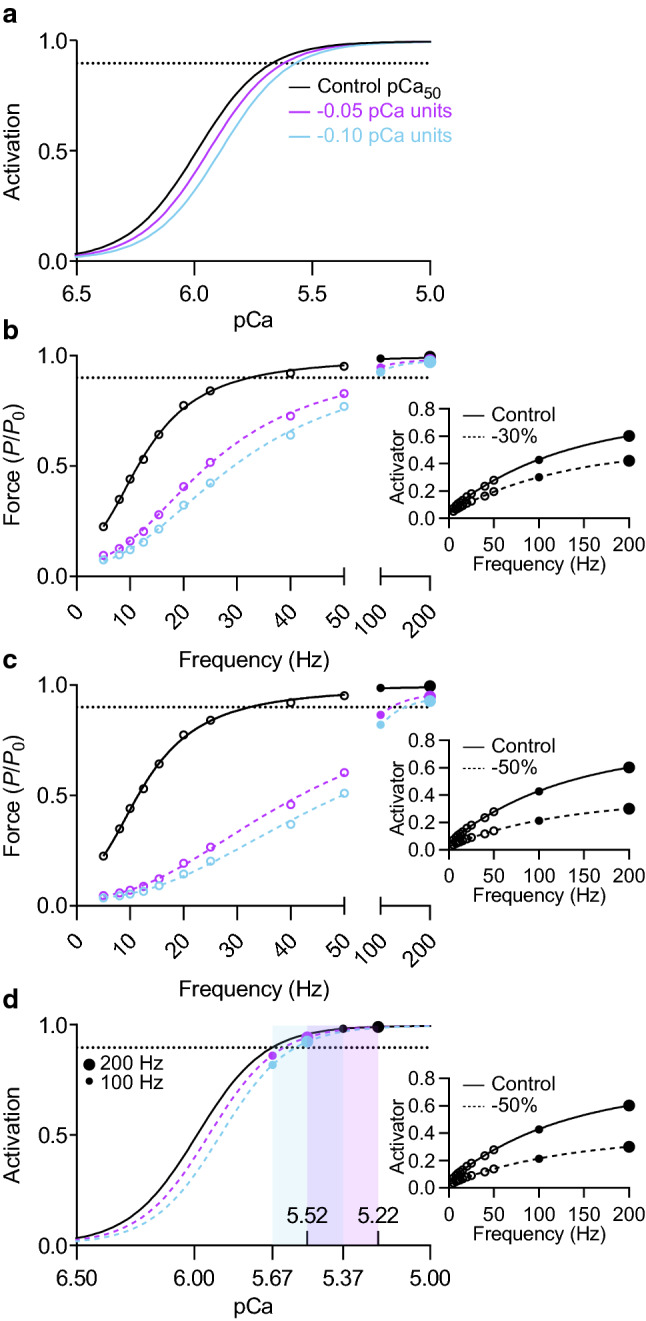
Effect of reducing calcium concentration and Ca^2+^ sensitivity in concert on maximal force generation. **a** Activation-pCa relationships with varying Ca^2+^ sensitivities. Ca^2+^ sensitivity was reduced by 0.05 and 0.10 pCa units relative to the control condition (5.99). **b***, ***c** Force-frequency relationships at 1.23 *L*_0_ for concomitant reduction in Ca^2+^ concentration and Ca^2+^ sensitivity. Force expressed relative to *P*_0_ of control condition. Instantaneous activator concentration was reduced by 30% in *b* and 50% in *c*. *Inset*, activator-frequency relationships for control and reduced Ca^2+^ concentration (dashed) conditions. **d** Activation-pCa relationships illustrating Ca^2+^ concentration and activation level for 100 (small circle) and 200 Hz (large circle) stimulation. Data are shown for a 50% reduction in instantaneous activator concentration. The two overlapping shaded regions represent the Ca^2+^ concentration range between the control and 50% reduction conditions for 100 and 200 Hz stimulation. *Inset*, activator-frequency relationships

Reducing Ca^2+^ concentration and Ca^2+^ sensitivity in concert lowered force dramatically at submaximal stimulation frequencies but only slightly reduced maximum tetanic force (Fig. 6b, c). Reducing Ca^2+^ availability by 30% and lowering pCa_50_ by 0.05 pCa units merely reduced the cross-bridge activation level to 98% during 200 Hz stimulation (Fig. 6b). Even when calcium concentration and calcium sensitivity were concurrently reduced by 50% and 0.10 pCa units, respectively, cross-bridge activation (*Act*) still exceeded 92% during 200 Hz stimulation (Fig. 6c). For 100 Hz stimulation, the steady-state Ca^2+^ concentration was 29% lower compared to 200 Hz. As such, there was a more significant reduction in cross-bridge activation level during 100 Hz stimulation when a 50% reduction in Ca^2+^ concentration was imposed and Ca^2+^ sensitivity was concurrently reduced by 0.05 (*Act* = 87%) or 0.10 pCa (*Act* = 82%) units (Fig. 6c). Figure 6d illustrates how the sigmoidal form of the force-pCa relationship limits the effect of an imposed reduction in Ca^2+^ concentration on maximum tetanic force, even when the Ca^2+^ sensitivity of force is reduced by 0.1 pCa units.

#### Slower Ca^2+^ uptake and lower Ca^2+^ release or lower Ca^2+^ sensitivity in concert

**Fig. 7:**
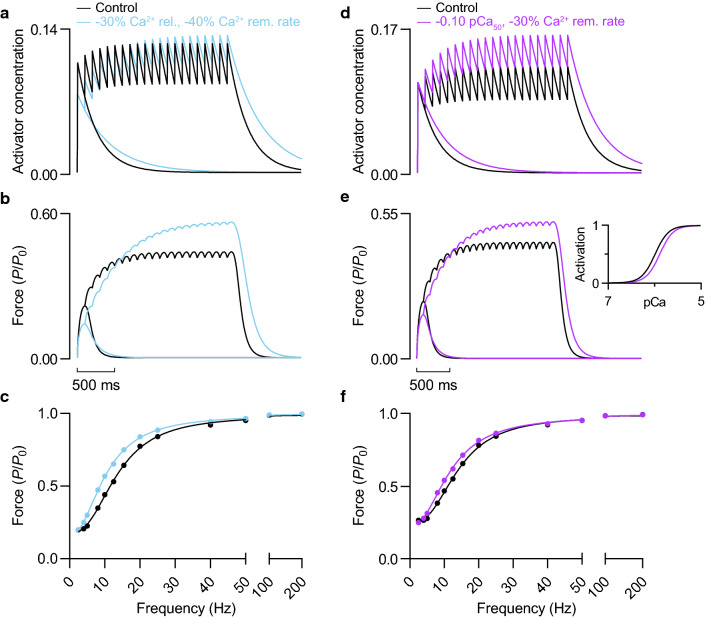
Effect of reducing Ca^2+^ release and Ca^2+^ uptake rate, or Ca^2+^ sensitivity and Ca^2+^ uptake rate in concert. **a**, **d** Activator concentration and **b**, **e** force during a twitch and sustained 10 Hz stimulation at 1.23 *L*_0_. In *a*, Ca^2+^ release was reduced by 30%, but the rate constant of Ca^2+^ uptake was also reduced by 40% (67% increase in *τ*_2_) relative to the control condition. In *d*, the rate constant of Ca^2+^ uptake was reduced by 30% (43% increase in *τ*_2_), but the Ca^2+^ sensitivity of force (*e inset*) was reduced by 0.10 pCa units relative to the control condition. **c**, **f** Force-frequency relationships at 1.23 *L*_0_. Force expressed relative to *P*_0_ of control condition

The pronounced deficit in steady-state force at low and moderate stimulation frequencies that resulted from a 30% reduction in Ca^2+^ release (Fig. 3e) was more than balanced by concurrently decreasing the rate constant for Ca^2+^ uptake by 40% (Fig. 7b, c). The reduction in Ca^2+^ release caused force to be lower at the beginning of the contraction, as is evident when twitch force is compared (Fig. 7b); however, the slower rate of Ca^2+^ removal allowed Ca^2+^ to accumulate to a higher steady-state concentration (Fig. 7a). A similar compensation effect occurred when Ca^2+^ uptake rate was reduced by 30% whilst Ca^2+^ sensitivity was lowered by 0.10 pCa units. During a twitch and at very low stimulation frequencies, lower Ca^2+^ sensitivity reduced force (Fig. 7e, f). At faster frequencies, the increase in Ca^2+^ concentration more than compensated for the reduction in Ca^2+^ sensitivity, shifting the force-frequency relationship leftward (Fig. 7e, f).

### CE-SEE interaction

#### Maximum velocity of shortening (***V***_max_)

**Fig. 8:**
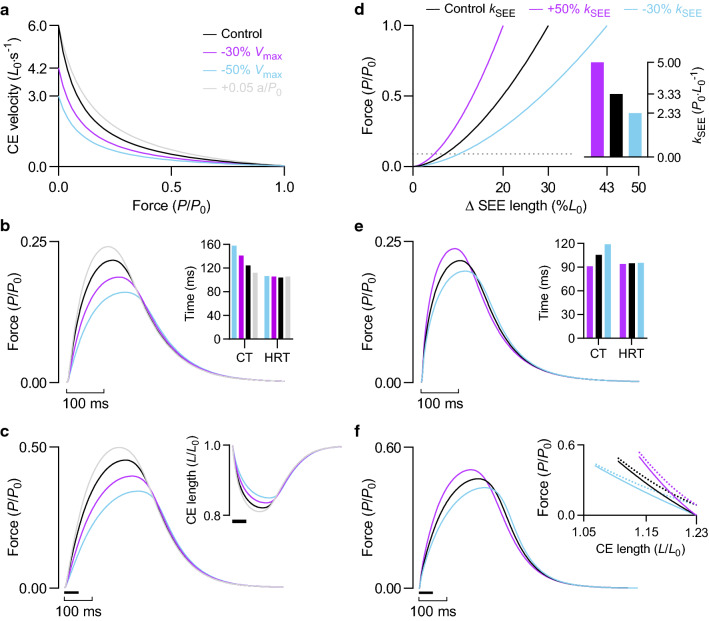
Effect of intrinsic shortening speed and SEE stiffness on force development. **a** Force–velocity relationships with varying values of *V*_max_ and a/*P*_0_. *V*_max_ was reduced by 30 and 50% relative to the control condition, and a/*P*_0_ was increased by 0.05 for illustrative purposes only. **d** SEE force–length relationships with varying stiffnesses. Deformation expressed as percentage of CE *L*_0_. Average SEE stiffness normalised to *P*_0_ and CE *L*_0_ (*k*_SEE_, inset) was reduced by 30% and increased by 50% relative to the control condition. Dotted line indicates passive force at optimal initial CE length of 1.23 *L*_0_. **b***, ***e** Twitch force at an initial CE length of 1.0 and 1.23 *L*_0_, respectively. *Inset*, twitch contraction time and half-relaxation time. **c**, **f** Force during a brief tetanic contraction (50 ms, 100 Hz) at an initial CE length of 1.0 and 1.23 *L*_0_, respectively. *Inset*, CE length as a function of time in *c* and force in *f*; the dotted lines indicate total force. Solid bar underneath force and CE length traces at contraction onset represents duration of stimulation

Because the CE shortens against the stretch of the SEE during force development (see Fig. 8c *inset*), reducing *V*_max_ by 30% reduced twitch force by 12% and increased twitch contraction time by 13%; twitch half-relaxation time was practically unaltered (Fig. 8b, *inset*). A similar effect was observed for a brief tetanic contraction (50 ms, 100 Hz) following a 30% reduction in *V*_max_ (Fig. 8c). Lowering *V*_max_ by 50% increased the loss of force and further prolonged the rise of force. Brief tetanic contractions were performed at an initial CE length of 1.0 *L*_0_, illustrating that the reductions in peak force with decreasing *V*_max_ arise despite more favourable final CE lengths—greater force arises from greater shortening against the stretch of the SEE (Fig. 8c *inset*). Increasing a/*P*_0_ produced qualitatively similar results for twitch and tetanic contractions as increasing *V*_max_ (Fig. 8).

#### SEE stiffness (***k***_SEE_)

Reducing SEE stiffness by 30% prolonged twitch contraction time by 13% and attenuated twitch force by 9%. Conversely, increasing SEE stiffness by 50% abbreviated twitch rise time by 14% and increased twitch force by 9% (Fig. 8e). The attenuation or improvement of the rate of force development and peak force during a brief contraction was not the result of less or more favourable CE operating lengths, which were restricted to the descending limb of the force–length relationship. For brief tetanic contractions performed at an initial CE length of 1.23 *L*_0_, peak force was greatest for the stiffer SEE condition, even though the average operating length of the CE was less favourable (Fig. 8f, *inset*).

#### ***V***_max_ & SEE stiffness interaction

**Fig. 9:**
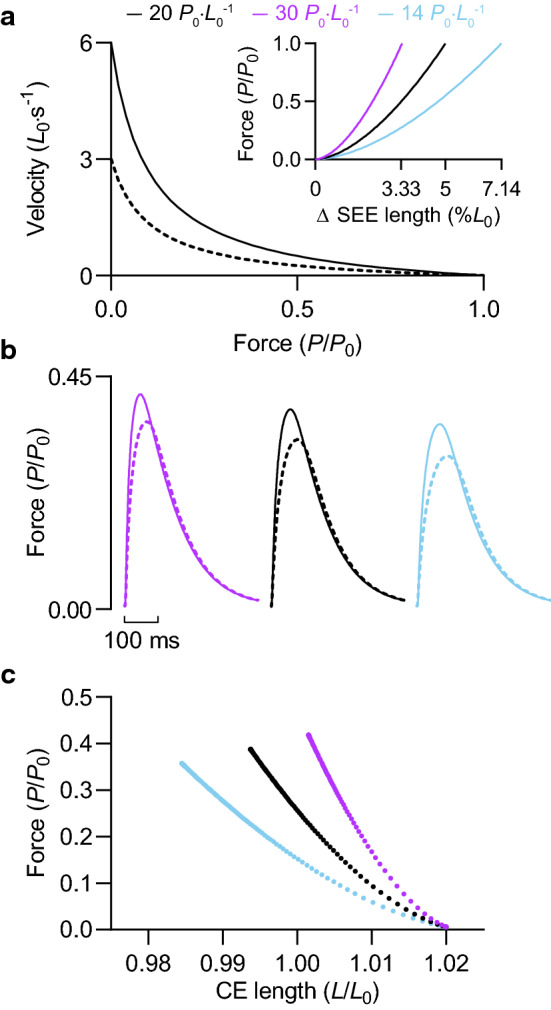
Effect of SEE stiffness on modulation of force development by *V*_max_. **a** CE force–velocity relationships for control condition and 50% reduction in *V*_max_ (dashed). *Inset*, SEE force–length relationships with varying stiffnesses. Normalised stiffness (*k*_SEE_) was reduced by 30% (14 *P*_0_·*L*_0_^−1^) and increased by 50% (30 *P*_0_·*L*_0_^−1^) relative to a reference *k*_SEE_ of 20 *P*_0_·*L*_0_^−1^, which gives a normalised SEE deformation value of 5%. **b** Twitch force at an initial CE length of 1.02 *L*_0_. **c** Force as a function of instantaneous CE length during a twitch. CE length was constrained to the plateau region of the CE force–length relationship

Decreasing *V*_max_ by 50%, from 6 to 3 *L*_0_·s^−1^, had a more modest effect on peak twitch force when SEE stiffness was adjusted to give normalised SEE deformations of less than 8% (compare Fig. 9b and 8b). At 3.33% (i.e., 30 *P*_0_·*L*_0_^−1^), twitch force was 13% (0.05 *P*_0_) lower (Fig. 9b). In contrast, reducing *V*_max_ by 50% lowered twitch force (at a comparable initial CE length) by 26% (0.06 *P*_0_) when normalised SEE deformation was 30% [i.e., 3.33 *P*_0_·*L*_0_^−1^ (Fig. 8b). The greater effect of intrinsic shortening speed on force development with decreasing SEE stiffness was independent of differences in force-generating potential related to CE length (Fig. 9c).

### Type I fibre fractional area

**Fig. 10:**
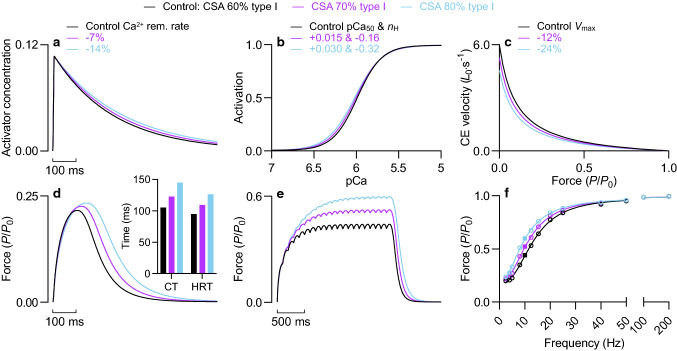
Effect of type I fibre fractional area. **a** Activator concentration during a twitch, **b** activation-pCa relationship, and **c** force–velocity relationship for varying fractions of muscle cross-sectional area (CSA) occupied by type I fibres (i.e., type I MHC content). Control values for Ca^2+^ removal (rem.) rate, pCa_50_ and *n*_H_, and *V*_max_ were adjusted after back-calculation of the fibre type specific value from a defined fibre type-related difference and a weighting factor proportional to the fractional area of type I fibres. The fractional area of type I fibres was increased by 0.10 and 0.20 from a control value of 0.60 (i.e., 60%). **d** Twitch force at an initial CE length of 1.23 *L*_0_. *Inset*, twitch contraction time and half-relaxation time. **e** Force during sustained 10 Hz stimulation at 1.23 *L*_0_. **f** Force-frequency relationship at 1.23 *L*_0_. Active force expressed relative to *P*_0_ of control condition. Solid squares denote the force for 10 Hz stimulation

Adjusting the rate constant of Ca^2+^ decay, the force-pCa relationship, and *V*_max_ to reflect a fractional increase in type I fibre area resulted in elevated twitch force and prolonged force rise and decay during a twitch (Fig. 10). Specifically, increasing the type I fibre area from 0.60 to 0.70 increased the twitch contraction time and half-relaxation time by 17 and 15%, respectively (Fig. 10d, *inset*). The slowing effect increased to 37 and 33% when type I fibre area was increased to 0.80. Both adjustments of type I fibre fractional area increased force at submaximal stimulation frequencies (Fig. 10e), with the latter causing the greatest leftward shift of the force-frequency relationship (Fig. 10f).

### Model optimisation to simulate plantar flexor twitch of old men

**Fig. 11:**
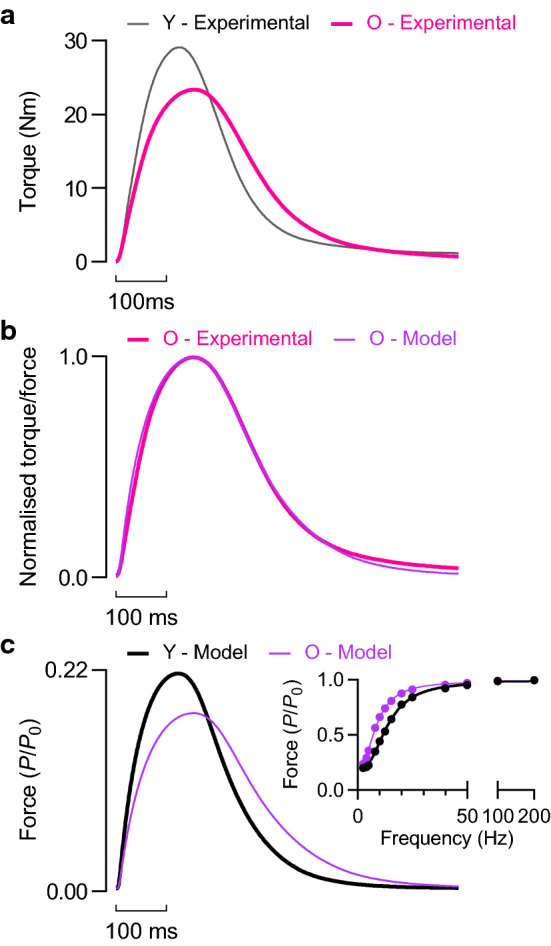
Model optimization to simulate time course and relative amplitude of plantar flexion twitch of old men. **a** Measured plantar flexion twitch torque of young (Y) and older (O) men. **b** Plantar flexion twitch torque of older men and simulated twitch force after parameter optimisation. Twitch contraction time and half-relaxation time for experimental and simulated data were identical following optimization (CT: 154 ms; HRT: 134.5 ms). **c** Simulated twitch force for young and old muscle. The relative deficit in twitch amplitude owing to ageing was 18% for simulated and experimental conditions. *Inset*, force-frequency relationships, where force is expressed relative to the respective *P*_0_ of each muscle

The plantar flexor twitch of older men was of a lower amplitude (18%) and exhibited a prolonged contraction time (25%) and prolonged half-relaxation time (29%) compared to young adult men (Table 2, Fig. 11a). The weaker, slower twitch in advanced age was well-simulated by the model with ageing-realistic adjustments to a few parameters (Fig. 11b, c). To emulate the time course and relative amplitude of the twitch exhibited by older men, which was possible with multiple parameter combinations, required significant slowing of Ca^2+^ uptake rate (~ 25%, Table 3). Because slowing the uptake of Ca^2+^ prolonged the duration of the Ca^2+^ transient, which increased twitch force, it was possible for the reduction in force-generating capacity (23%) to exceed that of the reduction in twitch force (18%) despite additional force loss from reductions in *V*_max_ and SEE stiffness (Table 3). A smaller reduction in force-generating capacity (15%) was possible with the addition of modest reductions in Ca^2+^ release and Ca^2+^ sensitivity, which had to be balanced by further slowing of Ca^2+^ uptake. Incorporating slower Ca^2+^ uptake produced a leftward shift of the force-frequency relationship (Fig. 11c *inset)*.

**Table 2:** Twitch properties of plantar flexors in young and older men

	Young	Older	*p* value	*η*^*2*^
Contraction time (ms)	123.6 ± 8.6	154.8 ± 24.7	< 0.001	0.50
Half-relaxation time (ms)	100.2 ± 17.2	129.0 ± 24.4	0.003	0.30
Torque (Nm)	29.3 ± 4.4	24.0 ± 5.2	0.011	0.22

Values represent the mean ± SD

**Table 3:** Comparison of optimized model parameters

	*τ*_1_	*τ*_2_	[a]_50_	pCa_50_	*n*_H_	*V*_max_	*k*_SEE_	FGC
	(s)	(s)		(pCa)		(*L*_0_·s^−1^)	*(P*_0_·*L*_0_^−1﻿^)	
Young	0.0422	0.256	0.1025	5.99	3	6	3.33	1.0
Old (a)	0.0422	0.339	0.1025	5.99	3	4.8	3.13	0.77
Old (b)	0.0431	0.345	0.1060	5.97	3	5	2.94	0.85

*FGC* force-generating capacity. Multiple solutions possible for Old muscle with varying FGC

## Discussion

Ca^2+^ transportation and calcium-activated force are perturbed in advanced age. Model simulations in the current work show that imposing literature-informed deficits in free Ca^2+^ concentration or Ca^2+^ sensitivity of force results in a substantial loss of submaximal force and a slow-to-fast shift in several indices of contraction speed. Their combined effect is especially dramatic. Imposing slowed Ca^2+^ reuptake had the opposite effect on contractile performance, increasing twitch force and the ratio of twitch-to-tetanic force, prolonging the duration of contraction, and shifting the force-frequency leftward. Simulations estimating the effect of a fractional increase in type I fibre area produced the same outcomes, although to a lesser extent. It is difficult to find support from human or animal studies for a slow-to-fast shift in contraction speed mediated by ageing in either single muscle fibres or whole muscle. Rather, the contractile properties of muscle in advanced age are understood to be defined by slowing (reviewed by Hunter et al., 1998, 2016; Larsson et al., 2018)—twitch contraction time and half-relaxation time are longer (e.g. Vandervoort and McComas, 1986), tetanic force decay is slower (e.g. Tevald et al., 2009), and the force-frequency relationship is shifted to lower frequencies (e.g. Brooks and Faulkner, 1988). As such, incorporating slower Ca^2+^ removal, or a combination of slower Ca^2+^ removal, greater Ca^2+^ sensitivity, lower cooperativity, and slower intrinsic shortening speed—to reflect an increase in type I fibre content—emulated many aspects of contractile performance frequently reported in advanced age.

### Ca^2+^ uptake

The extent to which the rise and decay of twitch force were prolonged by slowing Ca^2+^ removal was consistent with experimental observations of slowed twitch speed in advanced age (Vandervoort and McComas 1986; Brooks and Faulkner 1988; Larsson and Salviati 1989; Hicks et al. 1991; Alway 1995; Connelly et al. 1999). In fact, the predictions were comparable to experimental data of the association between slowed SR Ca^2+^ uptake activity and twitch speed (Narayanan et al. 1996). For the soleus muscle of old rats, a 52% deficit in SR Ca^2+^ uptake activity was accompanied by a 28 and 48% increase in twitch contraction time and half-relaxation time, respectively (Narayanan et al. 1996). When we imposed a 30% decrease in the rate constant for Ca^2+^ uptake, twitch contraction time and half-relaxation time increased by 25 and 38%. At 50%, slowing of the twitch, and the associated increase in submaximal force, far exceeded typical ageing-related slowing. To our knowledge, intracellular Ca^2+^ transients during twitches in young and old muscle have been compared in terms of amplitude but not half-width or rate of decay (González et al. 2003; Eshima et al. 2020). It would be advantageous for the model to incorporate the extent of slowing observed for the decay phase of the Ca^2+^ transient in a contracting fibre rather than the reduction in Ca^2+^ uptake rate demonstrated for an isolated SR vesicle or muscle homogenate.

Slower Ca^2+^ removal increased twitch force and, therefore, increased the ratio of twitch-to-tetanic force. Consistent with the model simulations, twitch force and twitch rise time are inversely related to the decay rate constant of the intracellular Ca^2+^ transient in single fibres (Sun and Edman 1996). Maintenance of twitch force despite a considerable deficit in maximum force or a higher ratio of twitch-to-tetanic force are commonly reported in advanced age (Carlsen and Walsh 1987; Pettigrew and Gardiner 1987; Hicks et al. 1991; van Schaik et al. 1994; Brown and Hasser 1996; Connelly et al. 1999; Klass et al. 2005; Moran et al. 2005). Slower Ca^2+^ removal, by prolonging the duration for which the contractile apparatus is exposed to Ca^2+^ during a twitch, may partially offset or completely compensate for intrinsic processes that facilitate force loss, such as lower free Ca^2+^ concentration and lower Ca^2+^ sensitivity.

For contractions at submaximal stimulation frequencies, slower Ca^2+^ removal led to greater steady-state Ca^2+^ availability, which resulted in higher forces and a leftward shift of the force-frequency relationship. An age-related shift of the force-frequency relationship toward lower frequencies is a common observation for both human (Narici et al. 1991; Roos et al. 1999; Allman and Rice 2004; Tevald et al. 2009) and animal skeletal muscle (Larsson and Edström 1986; Brooks and Faulkner 1988; Alway 1995; González et al. 2000; Moran et al. 2005). Generally, elevated force generation at submaximal frequencies is accompanied by an increase in twitch contraction time, half-relaxation time, or both. Because altered activation dynamics causing prolonged force rise and decay result in a slower fusion frequency, it is not surprising that aged muscles exhibiting normal twitch speed tend not to exhibit elevated relative force at submaximal frequencies (Walters et al. 1990; González et al. 2000; Dalton et al. 2010a; Elliott et al. 2016). Simulations incorporating a large reduction in Ca^2+^ sensitivity or moderate impairment of Ca^2+^ release in concert with slower Ca^2+^ uptake indicate that an ageing-appropriate increase in submaximal force may still be possible if these alterations coexisted. It seems less likely that a leftward shift of the force-frequency relationship would arise if SR Ca^2+^ release was greatly impaired, especially if the impairment occurred in concert with lower Ca^2+^ sensitivity or was only balanced by a modest slowing of SR Ca^2+^ uptake.

A limited number of studies have recorded intracellular Ca^2+^ transients in contracting fibres from young and old muscles, fewer have examined a twitch or employed a range of submaximal stimulation frequencies, and none appear to have studied slow twitch fibres or examined the decay of the intracellular Ca^2+^ transient (González et al. 2003; Andersson et al. 2011; Umanskaya et al. 2014; Eshima et al. 2020). Nonetheless, these studies support the view that impaired SR Ca^2+^ leads to lower free Ca^2+^ concentrations during both submaximal and maximal contractions. Therefore, because the free Ca^2+^ concentration reflects the net effect of Ca^2+^ release and removal processes, these observations suggest that impaired SR Ca^2+^ uptake is not a universal outcome, presents at a more advanced age with respect to impaired SR Ca^2+^ release, or is only capable of minimising the deficit in free Ca^2+^ concentration caused by impaired Ca^2+^ release. According to the model predictions, for slower contraction speed to arise in the presence of a lower free Ca^2+^ concentration, there would need to be considerable involvement from an alternative mechanism. Future work should be directed at establishing whether impaired SR Ca^2+^ release and slower SR Ca^2+^ uptake coexist, how they interact, or why submaximal force in advanced age isn’t disproportionately lower given the large deficit in free Ca^2+^ concentration and possible exacerbation by lower Ca^2+^ sensitivity.

### Type I fibre fractional area

Simulating an elevated fractional area of type I fibres also produced an appropriate level of slowing. For simulations incorporating a fractional increase of 0.1 or 0.2, the relative increases in twitch contraction time and twitch half-relaxation time, and of normalised force at submaximal stimulation frequencies, were similar to the age effect reported by some studies (Fitts et al. 1984; Davies et al. 1986; Roos et al. 1999; Connelly et al. 1999). Greater age-related prolonging of the contraction time and/or half-relaxation time, a more pronounced shift in the force-frequency relationship, or both (Vandervoort and McComas 1986; Brooks and Faulkner 1988; Alway 1995; Narayanan et al. 1996; Baudry et al. 2005; McNeil et al. 2005; Dow et al. 2005), may indicate that our weighting approach was not entirely effective or that the imposed fibre type differences were too conservative. Alternatively, the greater magnitude of slowing demonstrated by these studies may implicate an additive effect or the sole involvement of slower Ca^2+^ uptake (Narayanan et al. 1996); simulations of the latter produced larger effects.

Slowed SR Ca^2+^ uptake rate has not been consistently demonstrated in advanced age, at least not for rat muscles (Fitts et al. 1984; Larsson and Salviati 1989; Narayanan et al. 1996; Thomas et al. 2010; Russ et al. 2014). Comparatively, greater evidence can be found to support an elevated fractional area of type I fibres (Coggan et al. 1992; Kadhiresan et al. 1996; Cui et al. 2008; Elliott et al. 2016), especially for the human vastus lateralis muscle (Larsson et al. 1978; Klitgaard et al. 1990a; Hunter et al. 1999; Short et al. 2005; Korhonen et al. 2006; Nilwik et al. 2013; Lamboley et al. 2015; Brocca et al. 2017; Sonjak et al. 2019; Soendenbroe et al. 2022). In some instances, the fast-to-slow shift in MHC isoform content may manifest as a reduction in MHC IIb content and an increase in MHC IIa or hybrid MHC isoforms (Hepple et al. 2004; Cui et al. 2008). Myosin isoform composition correlates strongly with whole muscle performance (Ranatunga and Thomas 1990; Harridge et al. 1996). Slower contractile properties in advanced age have been associated with a greater fractional content of MHC I (Klitgaard et al. 1990a; Korhonen et al. 2006), demonstrated in the absence of slowed SR Ca^2+^ uptake (Larsson and Salviati 1989), and observed without slower Ca^2+^ removal being the rate-limiting process (Hunter et al. 1999). Our simulations add weight to this body of evidence—fibre-type related differences in contraction speed appear sufficient for a moderate-to-large age-related increase in type I fibre content to account for empirical observations of slowed whole muscle contraction speed.

### Ca^2+^ sensitivity and free Ca^2+^ concentration

Lower Ca^2+^ sensitivity of force and excitation-SR Ca^2+^ release decoupling are thought to play a role in the age-related decline of muscle specific force (Delbono et al. 1995; González et al. 2003; Andersson et al. 2011; Lamboley et al. 2015). The model predictions suggest that the deficits in free Ca^2+^ concentration and Ca^2+^ sensitivity reported in the literature, despite dramatically reducing force at submaximal stimulation frequencies, are insufficient to appreciably lower maximum tetanic force (< 5%). Our findings are supported by experimental observations from studies of dantrolene exposure (Krarup 1981; Macintosh et al. 2011) and low-frequency fatigue (Westerblad et al. 1993; Chin and Allen 1996; Glass et al. 2018; Olsson et al. 2020). For example, dantrolene partially inhibits SR Ca^2+^ release (Desmedt and Hainaut 1977), inducing moderate reductions in twitch force (27–53%) or shifting the force-frequency relationship rightward without appreciable, if any, tetanic force loss [0–6% (Krarup 1981; Macintosh et al. 2011)].

Of course, a greater deficit in maximum force would arise if the designation of thin filament activation (i.e., cross-bridge activation) during maximum tetanic stimulation was greatly overestimated in the model. This assertion would imply that maximum tetanic stimulation does not induce saturating Ca^2+^. However, our designation seems appropriate because tetanic force plateaus with increasing stimulation frequency despite an increasing free Ca^2+^ concentration (Westerblad and Allen 1993; Glass et al. 2018, 2020). Similarly, several studies demonstrate that tetanic forces with and without caffeine—which potentiates SR Ca^2+^ release—can be virtually identical, if not equal (Lannergren and Westerblad 1991; Westerblad and Allen 1991; Glass et al. 2018; Olsson et al. 2020).

Thin filament activation is also worth considering from the perspective of voluntary muscle excitation. Motor unit discharge rates during a maximal voluntary contraction are considerably lower than the stimulation rate required for muscle maximal tetanic force (Roos et al. 1999; Dalton et al. 2010a; Kirk and Rice 2016). Asynchronous stimulation, by minimising the oscillation of fibre length against series elasticity (Sandercock 2006), can elevate force at low and intermediate stimulation frequencies without reducing the frequency required for maximal tetanic force (Rack and Westbury 1969). As such, the discrepancy could infer submaximal thin filament activation during a volitional effort. Voluntary muscle activation isn’t easily quantified (Horstman 2009), and raising single fibre force from just 0.95 to 1.0 *P*_0_ can require a near two-fold increase in stimulation frequency and free Ca^2+^ concentration (Glass et al. 2018). If voluntary activation were submaximal, even only slightly, maximal voluntary contraction force would be lowered dramatically by a reduction in free Ca^2+^ concentration or Ca^2+^ sensitivity.

This notion must be viewed with caution, however, because Lind and Petrofsky (1978) found that the entire force-frequency relationship could indeed be shifted to lower frequencies through asynchronous stimulation. Thus, the differences between the two modes of excitation appear to be more complex than appreciated. Nonetheless, the force-frequency relationship of the model, where force is 0.95 *P*_0_ at 50 Hz, is generally consistent with relationships established for human muscle groups in vivo (Marsh et al. 1981; Davies et al. 1982; Roos et al. 1999; Allman and Rice 2004) and animal studies of predominantly slow muscle at physiological temperatures (Ranatunga 1982; Larsson and Edström 1986).

A more significant deficit in maximum tetanic force (8%) arose when free Ca^2+^ concentration and Ca^2+^ sensitivity were lowered in concert by 50% and 0.10 pCa units, respectively. However, these modifications represent the upper limit of the age effect for Ca^2+^ sensitivity and Ca^2+^ availability reported in the literature. It’s possible that moderate ageing-related reductions in Ca^2+^ availability or Ca^2+^ sensitivity may compromise tetanic specific force when Ca^2+^ sensitivity is lowered further by reducing fibre length (Stephenson and Williams 1982; Martyn and Gordon 1988; Balnave and Allen 1996), decreasing muscle temperature (Maughan et al. 1995; Debold et al. 2006; Nelson and Fitts 2014) or inducing fatigue, which also impairs SR Ca^2+^ release (Westerblad and Allen 1991, 1993).

Although there are concurrent measurements of free Ca^2+^ and force from intact single fibres (González et al. 2003), as well as combined measurements from single fibres and whole muscle (Andersson et al. 2011; Umanskaya et al. 2014), respectively, that implicate impaired SR Ca^2+^ release as an important determinant of the age-related deficit in specific force, it is unlikely that this mechanism is wholly responsible. Specific force remains lower for old compared to young intact single fibres after caffeine administration, which mitigates the age-related deficit in free Ca^2+^ concentration (González et al. 2003). Corroborating this finding are numerous studies using skinned fibres from young and old muscle (Lowe et al. 2002; D’Antona et al. 2003; Zhong et al. 2006; Yu et al. 2007; Kim and Thompson 2013; Hvid et al. 2013; Lamboley et al. 2015), some of which demonstrated a deficit in specific force of 25% or more (Thompson and Brown 1999; Frontera et al. 2000; Lowe et al. 2001; Ochala et al. 2007; Power et al. 2016; Brocca et al. 2017). These bodies of work, along with our findings, suggest that a substantial proportion of the deficit in tetanic specific force exhibited by intact single fibres, as well as whole muscle, is independent of lower tetanic free Ca^2+^ concentration. Although, as we have illustrated, the latter may exert a more considerable effect when accompanied by a moderate-to-large reduction in Ca^2+^ sensitivity.

### CE-SEE interaction

In addition to activation dynamics, force development is regulated by the intrinsic speed of shortening and the stiffness of the SEE being acted upon by the CE (Hill 1938; Edman and Josephson 2007). Incorporating ageing-related reductions in *V*_max_ and SEE stiffness slowed and prolonged the rise of force. During a twitch or brief tetanic contraction, both modifications also attenuated peak force. The effect on force development of a given reduction in intrinsic shortening speed depended on SEE stiffness, being more modest for low normalised SEE compliances (< 8%), suggesting that force rise during a twitch of a single fibre may not be appreciably affected by a slower *V*_max_. The current work supports the involvement of slower intrinsic shortening speed and higher SEE compliance as factors contributing to prolonged and slower force rise in advanced age, although consideration may need to be given to the muscle preparation. Elevated tendon stiffness, conversely, would likely act to offset factors causing force development to be slowed or twitch force to be attenuated.

The simulations with altered SEE stiffness are generally consistent with experimental work with added compliance (Hill 1951; Brown and Matthews 1960; Bawa et al. 1976; Mayfield et al.2016b) and where active shortening has been limited by means of a small muscle stretch (Hill 1949; Griffiths 1991; Sawicki and Roberts 2009; Mayfield et al. 2016a). Our findings also appear to be quantitively appropriate, although there is limited information to draw upon. We found that 33 (5 vs. 3.33 *P*_0_·*L*_0_^−1^) and 53% (5 vs. 2.33 *P*_0_·*L*_0_^−1^) reductions in normalised SEE stiffness reduced twitch force by 16 and 29%, respectively. Cat soleus twitch force was reduced by 35–40% (Bawa et al. 1976) following the addition of a spring that we estimate reduced the in-series stiffness by ~ 87% [isometric twitch force: ~ 5 N; spring stiffness: ~ 1.52 N·mm^-1^; tendon stiffness from spindle null method at 5 N: ~ 10 N·mm^-1^ (Rack and Westbury 1984)].

We found that twitch force and contraction time were similarly affected by increased SEE compliance. However, empirical measurements show that the reduction in force mediated by added compliance is more pronounced compared to the associated delay in peak force, and that the latter may not increase in proportion to the former (Hill 1951; Bawa et al. 1976; Mayfield et al. 2016b). Modest delays in peak force in response to a large amount of added compliance may relate to the effects of length or active shortening on factors such as cross-bridge kinetics (Fenwick et al. 2021), Ca^2+^ sensitivity of force (Stephenson and Williams 1982; Martyn and Gordon 1988), and force depression (Joumaa et al. 2012). With this observation in mind and the fact that imposing lower SEE stiffness prolonged the twitch contraction time by just 13%, it appears that this adaptation may not be an important determinant of twitch rise time.

Importantly, neither adaptation produced other facets of slowed contractile speed, such as slower force decay or elevated force at submaximal stimulation frequencies. Whilst the effect of added compliance on force decay is inconsistent and only modest (Bawa et al. 1976; Mayfield et al. 2016b), slower force decay should accompany a slower intrinsic shortening speed, especially at intermediate loads (Jones et al. 2006). Ignoring a dramatic change in SEE stiffness, which would bring force–length effects and possibly length-dependent Ca^2+^ sensitivity into play, a shift in the force-frequency relationship must arise from factors affecting Ca^2+^ concentration and calcium-activated force. Greater compliance and slower intrinsic shortening may minimise force oscillations or increase the apparent degree of fusion without affecting average force.

### Model simulation of age effect observed for experimental twitch data

The age-related prolonging of force rise and decay during a twitch observed for the plantar flexors in this study was within the range of slowing reported by others for the same muscle group (Davies et al. 1986; Vandervoort and McComas 1986; Simoneau et al. 2005; Dalton et al. 2009, 2010b). Simulating this age-related shift in twitch contraction speed was possible with parameter adjustments that fell within the ranges of age-related adaptation obtained from empirical observations used to inform earlier simulations. Specifically, the slower, weaker twitch was achieved with reductions in force-generating capacity (23%), Ca^2+^ uptake rate (24%), *V*_max_ (20%), and SEE stiffness (6%). Multiple solutions were possible, and it is likely that the slowing of twitch speed could have been emulated with parameter adjustments more consistent with an increase in type I fibre content. An increase in Ca^2+^ sensitivity and reduction in cooperativity would likely lessen the required reduction in Ca^2+^ uptake rate. We speculate because we imposed parameter limits that were consistent with the directionality of impairment reported in the literature.

## Summary

Age-related reductions in Ca^2+^ sensitivity and Ca^2+^ release abbreviated the twitch and dramatically lowered force during submaximal contractions (e.g., twitch, unfused tetanic contraction) without greatly influencing maximum tetanic force, even when acting in concert (< 10%). These predictions are at odds with experimental observations of the effect of age on indices of isometric contraction speed (i.e., twitch contraction time and half-relaxation time, force-frequency relationship), and suggest that reduced Ca^2+^ sensitivity and impaired SR Ca^2+^ release may contribute only modestly to the reduction in specific force in advanced age (depending on Ca^2+^ saturation during tetanic stimulation). Conversely, simulations that incorporated slowed Ca^2+^ removal or a greater fractional area of type I fibres prolonged the rise and decay of twitch force and shifted the force-frequency relationship leftward. These predictions are consistent with the characteristic fast-to-slow shift in contractile performance associated with ageing. Slowed and prolonged force development, and lower twitch force, also resulted from imposing a slower intrinsic shortening speed and lower SEE stiffness but occurred without a concomitant slowing of force decay or elevation of submaximal force. As such, these properties alone did not produce the characteristic slowing of contraction speed. The effect of *V*_max_ depended on SEE stiffness, and empirical observations do not always support a pronounced delay in peak force from added compliance, possibly because of additional factors related to active shortening not captured in the model. Simulating the slower, weaker twitch observed experimentally for the plantar flexors of older men required significant slowing of Ca^2+^ uptake (~ 25%) and could be coupled with an appreciable reduction in force-generating capacity (i.e., the reduction in force-generating capacity exceeded that of the deficit in twitch force). Slowed Ca^2+^ removal, when acting in concert, negated the depressive effects of moderate and large reductions in Ca^2+^ release and Ca^2+^ sensitivity, respectively.

## Conclusion

Whole muscle contractile performance in advanced age is characterised by slowed isometric contraction speed. This work provides support for the involvement of multiple mechanisms, although these adaptations do not necessarily affect the same aspects of contraction speed. As such, identifying the most important adaptations should be aided by characterising an array of isometric contractile properties in advanced age. Both slower Ca^2+^ uptake and a greater fractional area of type I fibres seem to be suitable mechanisms for explaining the slower isometric contraction speed exhibited by aged muscle. Simulations incorporating these adaptations with a degree of impairment reported in the literature generated realistic age-related changes in contractile behaviour. Ageing-appropriate adjustments to these parameters also emulated the age-related slowing of twitch speed observed experimentally. In general, the model simulations were well-supported by empirical observations. We propose that this model or similar models might be effective in determining a meaningful impairment threshold or identifying the factors contributing to altered contractile properties.

Adaptation of muscle function and structure in advanced age is inconsistent and wide-ranging, thus, careful consideration should be given to the strength of evidence implicating the presence of a particular adaptation (e.g., fibre type, species, activity level, age). With the multifaced nature of impairment in mind, we adjusted multiple model parameters concurrently and have illustrated the importance of considering interaction effects. Inconsistent reports regarding the effect of age on contractile performance may relate to variation in the disruption of function and structure. We believe this work underscores the utility of simple, yet physiologically-grounded and parameter rich Hill-type muscle models for studying conditions that involve a multitude of adaptations (e.g. ageing, disuse, training). Such models also hold great value in being able to predict functional performance (e.g. walking, standing from a chair) when used in musculoskeletal simulations (e.g. Song and Geyer 2018; Ong et al. 2019).

## Data Availability

The Hill-type muscle model developed in Simulink and a MATLAB application that can be used to run simulations are accessible as open source (under the MIT license) from GitHub [https://github.com/dlmayfield/Muscle-Model (Mayfield and Lichtwark 2022)]. Experimental twitch data from young and older human adults are accessible from this repository.
